# Investigations on the Role of N_2_:(N_2_ + CH_4_) Ratio on the Growth of Hydrophobic Nanostructured Hydrogenated Carbon Nitride Thin Films by Plasma Enhanced Chemical Vapor Deposition at Low Temperature

**DOI:** 10.3390/ma10020102

**Published:** 2017-01-24

**Authors:** Noor Hamizah Khanis, Richard Ritikos, Shafarina Azlinda Ahmad Kamal, Saadah Abdul Rahman

**Affiliations:** Low Dimensional Materials Research Centre, Department of Physics, Faculty of Science, University of Malaya, Lembah Pantai, Kuala Lumpur 50603, Malaysia; shafarinaazlinda@gmail.com (S.A.A.K.); saadah@um.edu.my (S.A.R.)

**Keywords:** chemical vapor deposition, carbon nitride, nanostructures, hydrophobic

## Abstract

Nanostructured hydrogenated carbon nitride (CN_x_:H) thin films were synthesized on a crystal silicon substrate at low deposition temperature by radio-frequency plasma-enhanced chemical vapor deposition (PECVD). Methane and nitrogen were the precursor gases used in this deposition process. The effects of N_2_ to the total gas flow rate ratio on the formation of CN_x_:H nanostructures were investigated. Field-emission scanning electron microscopy (FESEM), Auger electron spectroscopy (AES), Raman scattering, and Fourier transform of infrared spectroscopies (FTIR) were used to characterize the films. The atomic nitrogen to carbon ratio and sp^2^ bonds in the film structure showed a strong influence on its growth rate, and its overall structure is strongly influenced by even small changes in the N_2_:(N_2_ + CH_4_) ratio. The formation of fibrous CN_x_:H nanorod structures occurs at ratios of 0.7 and 0.75, which also shows improved surface hydrophobic characteristic. Analysis showed that significant presence of isonitrile bonds in a more ordered film structure were important criteria contributing to the formation of vertically-aligned nanorods. The hydrophobicity of the CN_x_:H surface improved with the enhancement in the vertical alignment and uniformity in the distribution of the fibrous nanorod structures.

## 1. Introduction

Nitrogen incorporation in carbon materials induces structural transformation of C–N, C-sp^3^ and C-sp^2^ bonding in compounds, and enhances their electronic characteristics [1,2]. Hydrogenated carbon nitride (CN_x_:H) nanostructures are being extensively studied as a suitable candidate for various applications, such as field electron emitters [3], visible-light photocatalysts [4], field effect transistors and probe tips [5], fuel cells [6], and sensors [7,8,9]. However, recent interest in the wetting properties of carbon-based nanostructures has created a new direction in the application of CN_x:_H materials [10,11]. A strategy for fabricating CN_x_:H nanostructured materials with hydrophobic surfaces is important, as these materials will steer potential applications in the cosmetics, bio-medical, semiconductor, and painting and printing industries [12,13].

Various synthesis approaches have been devised to produce these compounds, such as sputtering [14], arc discharge [15], laser ablation [16], and several chemical vapor deposition (CVD) techniques including hot wire CVD, electron cyclotron CVD, and radio frequency plasma enhanced CVD (PECVD) [17,18,19]. Typically, the 1-D structures produced by the catalytic growth process require high deposition temperatures, which can introduce contamination in the structures, and is not cost effective compared to catalyst-free and low-temperature deposition process. We have taken advantage of the large area, low-temperature deposition, easy handling, and parameter manipulation of PECVD to produce catalyst-free growth of carbon nanorods induced by nitrogen incorporation, as described in our earlier works. The formation of vertically-aligned carbon nanorods was shown to require a long deposition time, and was correlated to an increase in nitrogen incorporation and a significant presence of isonitrile bonds [20].

In this work, we report the effects of different nitrogen flow-rate to total gas flow-rate ratios, [N_2_:(N_2_ + CH_4_)] on the morphological, structural, and wetting properties of nanostructured hydrogenated carbon nitride (CN_x_:H) films grown by PECVD. The atomic nitrogen to carbon ratio and sp^2^ bonds in the film structures varied with facile adjustment of N_2_:(N_2_ + CH_4_) ratio. Important microstructural and bonding properties, which are crucial to the formation of nanostructures in the films, were identified even at small changes of the N_2_:(N_2_ + CH_4_) ratio. The influence of the morphology of the nanostructures formed on the wetting properties of the films will also be investigated.

## 2. Experimental Methods

Carbon nitride (CN_x_:H) nanostructured films were fabricated in an in-house designed radio frequency (rf-PECVD) system with a parallel-plate electrode configuration. Methane and nitrogen gases were used as the precursor gas source. The films studied in this work were deposited at different ratios of N_2_ to the total gas flow rate [N_2_:(N_2_ + CH_4_)] varying from 0.40 to 0.90. The films were grown on bare (111) p-type Si substrates at a rf power of 80 W. The chamber (Kejuruteraan Wing Hung, Kuala Lumpur, Malaysia) was evacuated to a base pressure of approximately 3 × 10^−5^ mbar. The substrate temperature was initially set at 100 °C, but intense plasma ion bombardments on the film surface increased the temperature to a saturation value of 220 °C within the deposition process of 60 min. Prior to the actual growth, the substrates were treated in hydrogen plasma (Kejuruteraan Wing Hung) for 10 min at gas flow rate, rf power, and deposition pressure of 50 sccm, 60 W, and 0.8 mbar, respectively.

Surface and cross section images of the CN_x_:H nanostructures were obtained by field emission scanning electron microscope (FESEM) using a FEI Quanta 200 FESEM (FEI Company, Hillsboro, OR, USA). A Jeol JAMP-9500F Field Emission Auger Microprobe (JEOL USA, Inc., Peabody, MA, USA) was employed to investigate the elemental composition of these films. The Raman spectra were measured using Horiba Jobin Yvon 800 UV Micro-Raman Spectrometer (HORIBA Scientific, Paris, France) at an excitation wavelength of 514.5 nm. A Perkin-Elmer System 2000 Fourier transform infrared (FTIR) (PerkinElmer, Waltham, MA, USA) spectrometer was used to determine the chemical bonding present in the films in the transmission mode. A contact angle (CA) measurement system (Theta Lite, Biolin Scientific, Stockholm, Sweden) equipped with SCA20 U software (DataPhysics Instruments GmbH, Filderstadt, Germany) was employed to measure the water contact angle of the CN_x_:H thin films in air at ambient temperature. Three microlitres of distilled water droplets were gently dropped onto the surface. Several measurements were done at different spots on the film surface, and the average CA value was taken as the contact angle value.

## 3. Results and Discussion

### 3.1. Field Emission Scanning Electron Microscopy

**Figure 1:**
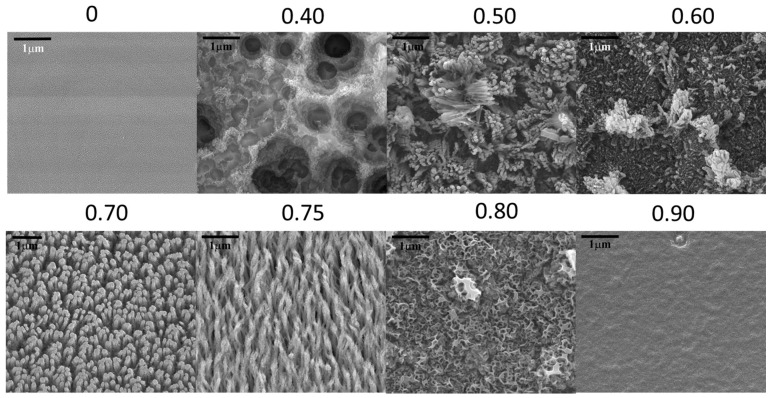
Field emission scanning electron microscopy (FESEM) surface images of CN_x_:H nanorods obtained at various N_2_:(N_2_ + CH_4_) flow rate ratios.

The surface morphology of the films deposited at different N_2_:(N_2_ + CH_4_) ratios obtained from FESEM is shown in Figure 1. The film deposited from pure methane (N_2_:(N_2_ + CH_4_) = 0) showed a smooth surface morphology. Fibrous nanorod structures were formed on the surface of the films deposited at N_2_:(N_2_ + CH_4_) ratios of 0.50–0.75. For the films deposited at N_2_:(N_2_ + CH_4_) ratios of 0.50 and 0.60, the nanorod structures formed in random in directions, clustered in bundles and scattered over the entire film surface. However, for the films deposited at N_2_:(N_2_ + CH_4_) ratios of 0.70 and 0.75, the nanorod structures formed in a more orderly fashion, and were uniformly distributed. Worm-like nanostructures were formed in the films deposited at N_2_:(N_2_ + CH_4_) ratio of 0.80. Nano-structures were not formed in the film deposited at N_2_:(N_2_ + CH_4_) ratio of 0.90, and a smooth, uniform morphology was observed. These images suggest that N_2_:(N_2_ + CH_4_) ratios of 0.50 to 0.75 are favorable for the formation of fibrous nanorod structures.

**Figure 2:**
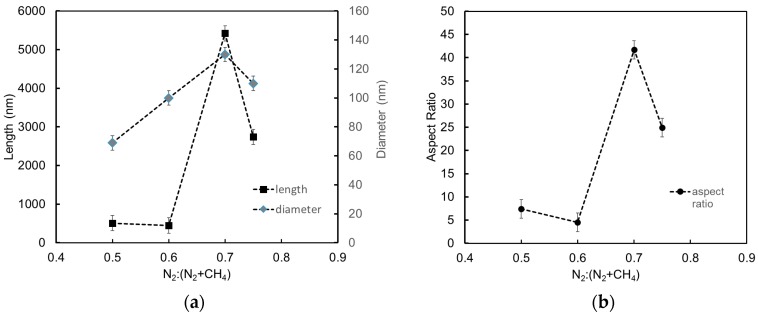
Variation of fiber length and diameter (**a**), and aspect ratio (**b**) with N_2_:(N_2_ + CH_4_).

**Figure 3:**
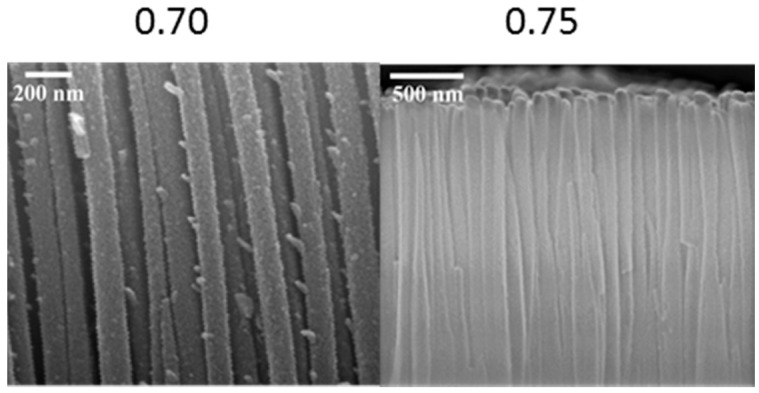
FESEM cross-section images for nanorod structures grown at N_2_:(N_2_ + CH_4_) flow rate ratio of 0.70 and 0.75. Selected magnifications of the images were used for clarity.

Figure 2 shows the variation of fiber length, diameter, and aspect ratio of the fibrous nanorods with N_2_:(N_2_ + CH_4_) ratio. The nanorod structures in the films deposited at N_2_:(N_2_ + CH_4_) = 0.70 and 0.75 showed significantly higher aspect ratios relative to the other two films. The cross-sectional images of these nanostructures are shown in Figure 3. Image magnification was selected in order to compare the structures. The vertically-aligned fibrous nanorods in these films grew directly from the surface of the films. The fibrous nanorods in the films deposited at N_2_:(N_2_ + CH_4_) ratio of 0.75 were vertically aligned, while those formed in the films deposited at N_2_:(N_2_ + CH_4_) ratio of 0.70 were slightly tilted. The length of the fibrous nanorods in the films deposited at a N_2_:(N_2_ + CH_4_) ratio of 0.70 was almost double those formed in the films deposited at N_2_:(N_2_ + CH_4_) ratio of 0.75, thus giving these nanostructures a higher aspect ratio.

### 3.2. Auger Electron Spectroscopy

**Figure 4:**
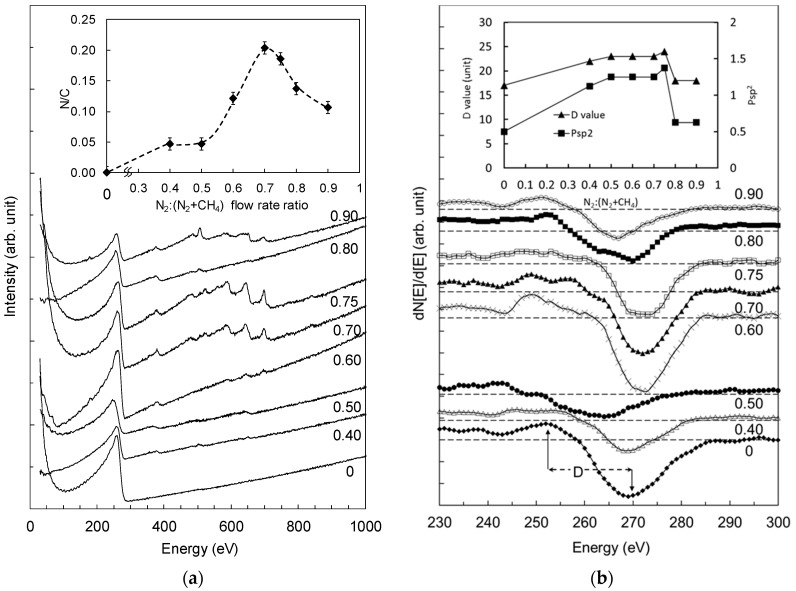
The (**a**) Auger electron spectroscopy (AES) and (**b**) KLL first derivative spectra of films deposited at different N_2_:(N_2_ + CH_4_) ratios. The inset in (**a**) is the variation of N/C ratio, and the inset in (**b**) is the variation of *D* values with N_2_:(N_2_ + CH_4_) ratio.

One of the most interesting aspects of this study is the narrow range of N_2_:(N_2_ + CH_4_) ratios within which the vertically-aligned fibrous nanorod structures were formed. It is therefore important to investigate how N_2_:(N_2_ + CH_4_) ratio influences the incorporation of nitrogen and the bonding configuration in the material leading to the formation of these fibrous nanorod structures. The Auger electron spectroscopy (AES) and the corresponding first derivative spectra of the films deposited at different N_2_:(N_2_ + CH_4_) ratio are shown in Figure 4a,b respectively. The peaks contributed by carbon and nitrogen in the energy range of 170–300 eV and 330–410 eV, respectively, are present in all AES spectra of the films. Films deposited at N_2_:(N_2_ + CH_4_) ratios of 0.7, 0.75, and 0.9 showed small peaks demonstrating a certain amount of Fe and Al metal contamination, which is believed to originate from the sputtering of the electrode material during the deposition. Since these peaks are also present in the AES spectrum of the film without nanostructures deposited at N_2_:(N_2_ + CH_4_) ratio of 0.9 (as shown in Figure 1), we believe that these metals did not act as the catalyst for the formation of these fibrous nanorod structures.

The nitrogen-to-carbon N/C ratio (shown in the inset of Figure 4a) increases to its maximum for the film deposited at N_2_:(N_2_ + CH_4_) ratio of 0.70, and decreases with further increase in the N_2_:(N_2_ + CH_4_) ratio. The vertically aligned-fibrous nanorod structures were formed in the films with the highest N/C ratios of 0.21 and 0.20, where the N_2_:(N_2_ + CH_4_) ratios were 0.70 and 0.75, respectively.

The first derivative of the AES spectra was further used to study the role of sp^2^ and sp^3^ bonds in the formation of the fibrous nanorod structures in the films. As shown in Figure 4b, the *D* value was determined from the difference in the value of the maximum and minumum points of the carbon KLL peak [21]. This value varies linearly with the sp^2^/sp^3^ ratio in the sample. The probabilities of finding sp^2^-C bonds in the film structure (Psp^2^) for pure graphite and diamond of 1 and 0, respectively, are used in the calibration to represent sp^2^-C bond content in the film. Consequently, the probability of finding sp^3^-C bond content in the film structure is (1 − *Psp*^2^). Using the standard calibration for *D* values of approximately 21 and 13–14 for graphite and diamond, respectively [22], as reported by Turgeon and Paynter [21], *Psp*^2^ in the film structure is derived where

(1) $$Psp^{2}=\frac{D-13}{8}$$

The plots of the *D* values and $Psp^{2}$ versus N_2_:(N_2_ + CH_4_) are shown in the inset in Figure 4b. Two different ranges of *D* values were obtained, corresponding to the differences in structure formation. The films with fibrous nanorod structures deposited at N_2_:(N_2_ + CH_4_) ratios of 0.50–0.75 show *D* values of 22–24. The other films deposited at N_2_:(N_2_ + CH_4_) ratios of 0.8–0.9 and from pure CH_4_—which have no fibrous nanorod structures—show *D* values of 16–18. The films with *Psp*^2^ values exceeding 1 are observed to result in the formation of fibrous nanorod structures emerging from the surface. This can be explained by the fact that the film is saturated with sp^2^ bonds, and the fibrous nanorod structures are actually precipitations of sp^2^ bonds in the film structure protruding out from the surface in the form of fibrous graphitic nanorods.

### 3.3. Raman Spectroscopy

**Figure 5:**
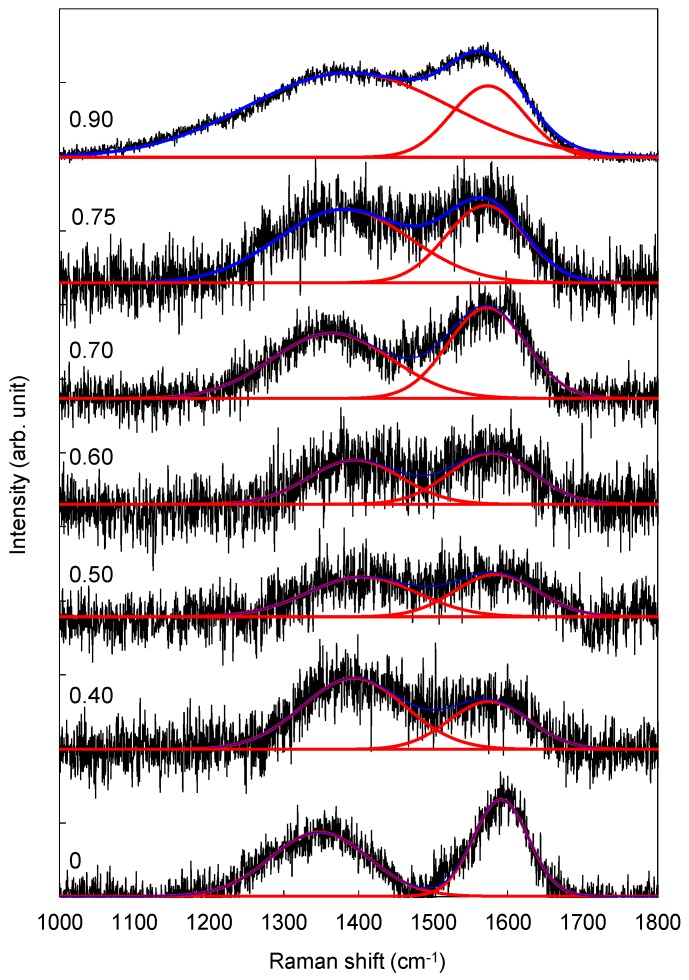
Raman scattering spectra of nanostructured CN_x_:H films prepared at different N_2_:(N_2_ + CH_4_) flow rate ratio. The deconvolution using Gaussian fittings are also shown.

The sp^2^ contribution in the formation of fibrous nanorod structures was further examined with visible-Raman scattering studies. The Raman spectra of the nanostructured CN_x_:H films deposited at various N_2_:(N_2_ + CH_4_) ratios are shown in Figure 5. The spectra consist of broad overlapping peaks of the G and D bands, similar to those usually observed for disordered carbon films. The D band is attributed to the breathing modes of A_1g_ symmetry involving phonons near the K zone boundary, and is usually related to the amount of disordered or imperfect sp^2^-C. The G band corresponds to the zone-centred E_2g_ mode of graphite banding, and is usually associated with pairs of sp^2^ atoms in both rings and chains [23].

**Figure 6:**
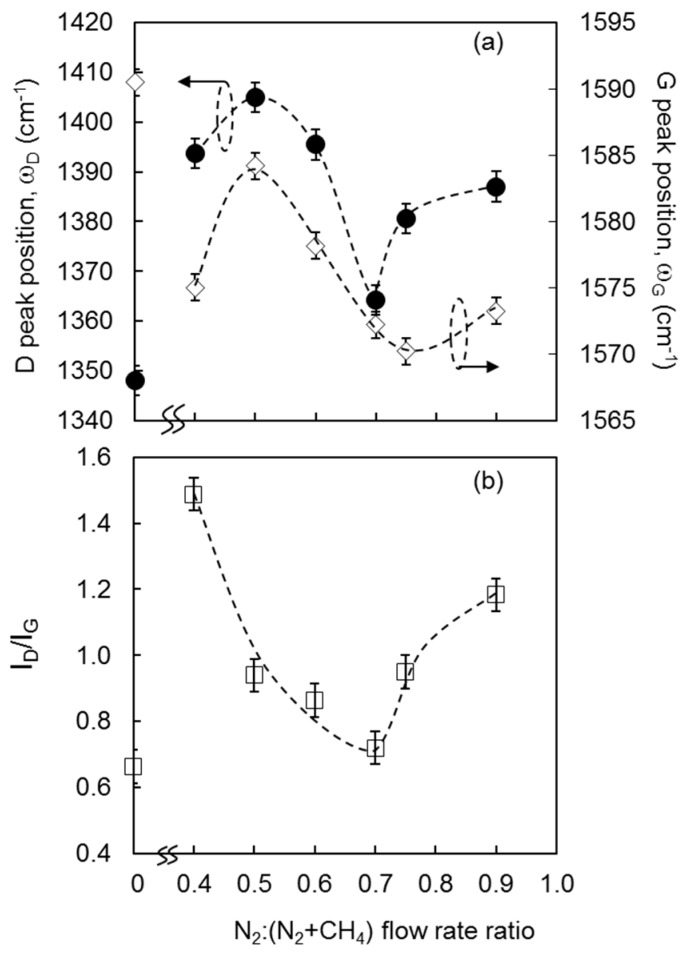
The variation of ωD (●) and ωG (◊) (**a**); and I_D_/I_G_ (□) (**b**) as a function of N_2_:(N_2_ + CH_4_) flow rate ratio.

The variations in the calculated band-position ωD for the D bands and ωG for the G bands, and D to G intensity ratios, I_D_/I_G_ with N_2_:(N_2_ + CH_4_) ratios are shown in Figure 6. I_D_/I_G_, ωD, and ωG of the films decreased to a minimum at N_2_:(N_2_ + CH_4_) ratio of 0.70. The decrease in I_D_/I_G_ indicates a decrease in the size of sp^2^-graphitic clusters, and a decrease in defects in the film structures [24]. This is supported by the red-shift in the ωD, which indicates a decrease in the number of ordered rings [24,25,26]. Similarly, the red-shift in the G band indicates an increase in the number of non-sixfold rings in the sp^2^-bonded carbon clusters [27]. All of these effects collectively reduce residual stress in the film structure, and contribute to the formation of uniformly distributed fibrous nanorod structures.

### 3.4. Fourier Transform Infrared Spectroscopy

**Figure 7:**
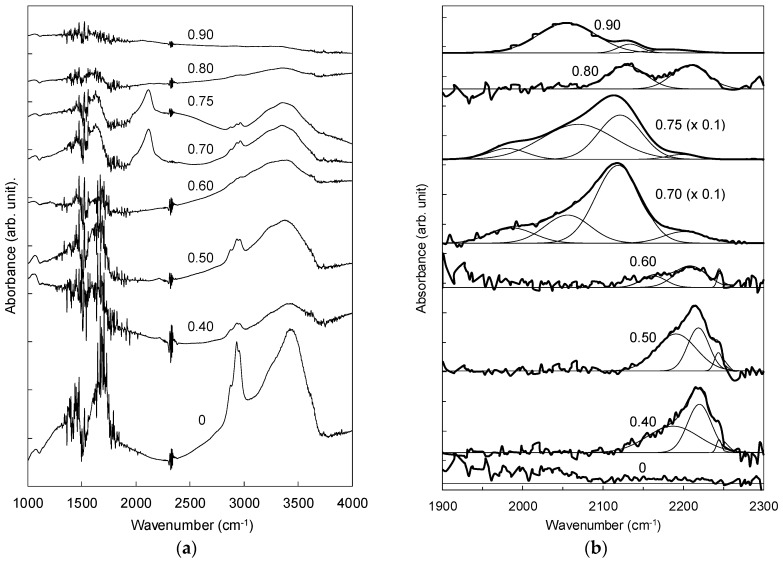
Variation of Fourier transform of infrared spectroscopy (FTIR) in the range of (**a**) 1000–4000 cm^−1^ and (**b**) 1900–2300 cm^−1^. The deconvolution for nitrile and isonitrile bonds are shown in (**b**).

Figure 7a presents the FTIR spectra of nanostructured CN_x_:H films deposited at different N_2_:(N_2_ + CH_4_) ratios. The absorption bands in these spectra are categorized into four main regions: (I) a range of 1300–1800 cm^−1^, which corresponds to the sp^2^ phases that includes the C=C and/or C=N, and N–H bonds; (II) a range of 1800–2200 cm^−1^, which reveals the presence the sp^1^ phase associated with the C≡N groups; (III) a range of 2800–3000 cm^−1^ which corresponds to the hydrogen-related sp^3^ phases of C–H groups; and (IV) a range of 3000 cm^−1^ and 3800 cm^−1^, which is assigned to the hydroxyl N–H and/or O–H groups [28,29,30]. Absorptions bands in regions (I), (III), and (IV) are observed in the spectra of all films, showing variations in peak intensity, indicating that the N_2_:(N_2_ + CH_4_) ratio has a strong influence on the bonding structures represented by these bands in these regions. However, detailed investigations were done in region (II) due to the significant presence of the band representing the C≡N bonding group in the films deposited at N_2_:(N_2_ + CH_4_) ratios of 0.70 and 0.75, which have a morphology of uniform growth of vertically-aligned fibrous nanorod structures, as shown in Figure 1.

The spectra in region (II) are deconvoluted into the component regions by employing standard Gaussian fitting, as shown in Figure 7b. There are five component bands in this region: bands which are attributed to both nitrile (−C≡N) and isonitrile (−N≡C) bands [29,31]; isolated and/or fused aromatic rings bonded either to isonitrile (−N≡C) at 2105 cm^−1^ or nitrile (−C≡N) at 2215 cm^−1^; hydrocarbon molecules bonded to isonitrile (−N≡C), including C_2_H_5_ and CH_3_ at 2160 cm^−1^ and 2190 cm^−1^, respectively; and wavenumber of 2245 cm^−1^, associated with the hydrocarbon groups (CH_3_, C_2_H_5_, etc.) bonded to nitrile (−C≡N). The absence of absorption bands within this region in the spectra of the film deposited from pure CH_4_ shows that the bands in this region are characteristic of N incorporation into the carbon network of the film structure. The films deposited at N_2_:(N_2_ + CH_4_) ratios of 0.40–0.60 show the presence of bands corresponding to bonding configurations of CH_3_ bonded to isonitrile (−N≡C) at (2180 ± 10) cm^−1^, isolated and/or fused aromatic rings bonded to nitrile (−C≡N) at (2216 ± 5) cm^−1^, and the hydrocarbon groups (CH_3_, C_2_H_5_, etc.) bonded to nitrile (−C≡N) at (2245 ± 5) cm^−1^

The dominant peak in this region shifts to (2115 ± 10) cm^−1^ for the films deposited at N_2_:(N_2_ + CH_4_) ratios of 0.7 and 0.75, which is associated with isolated and/or fused aromatic rings bonded to isonitrile. This peak is flanked by two key peaks at (2060 ± 7) and (2216 ± 5) cm^−1^, associated with diffused HCN [32] and isolated and/or fused aromatic rings bonded to nitrile bonds, respectively. The presence of aligned vertical fibrous nanorod structures in these films shows that these bonds are crucial in forming these nanostructures. This agrees with the results obtained from analysis of the D value and analysis done on AES data, where the saturation of sp^2^ bonds in the film structure contributed to the formation of the vertically-aligned nanostructures. The formation of long ordered networks is attributed to isonitrile bonds within the nanostructure, which have a tendency to form rigid, linear, and continuous non-terminating bonds. The presence of partial charge (−N^+^≡C^−^) bonds within the nanostructure strands contributes to the formation of vertically-aligned nanorod structures due to the interaction of these polarized bonds with the electric field created by the rf plasma.

### 3.5. Contact Angle Measurement

**Figure 8:**
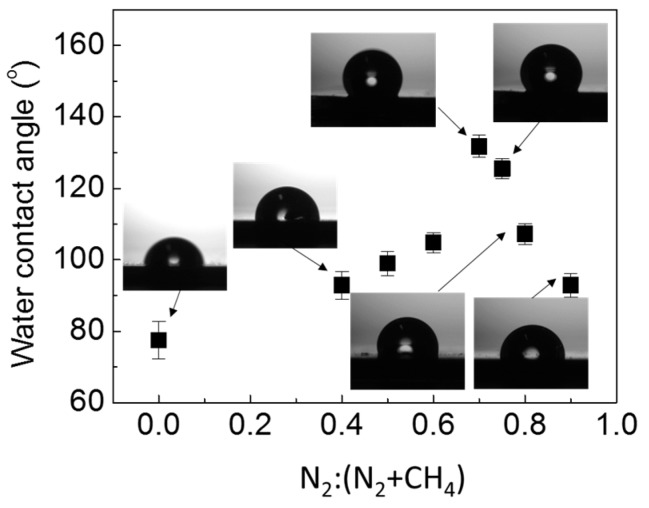
Variations in the contact anglefor CN_x_:H nanostructured films deposited at different N_2_:(N_2_ + CH_4_) flow rate ratios.

**Figure 9:**
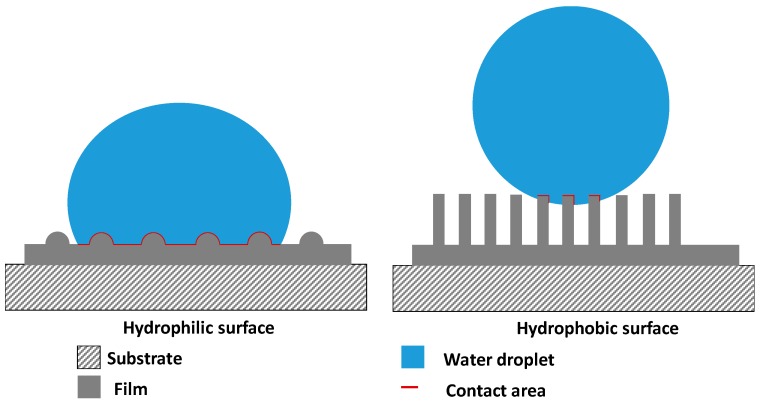
Film–water interaction for solid and nanostructured CN_x_:H films.

The contact angle (CA) measurements of the films as a function of N_2_:(N_2_ + CH_4_) ratios are shown in Figure 8. The film deposited from pure methane without nitrogen exhibited hydrophilic behavior, with a CA value of 77.5°. The CA increased almost linearly with N_2_:(N_2_ + CH_4_) ratio, and shifted towards hydrophobic behavior for the films deposited at N_2_:(N_2_ + CH_4_) ratios of 0.7 and 0.75. These two films demonstrated hydrophobic behavior, with CAs of 131.7° and 125.5° for N_2_:(N_2_ + CH_4_) ratios of 0.70 and 0.75, respectively. With further increase in N_2_:(N_2_ + CH_4_) ratio to 0.80 and 0.90, the CA value decreased, and the surface reverted back towards a hydrophilic behavior for the film deposited at N_2_:(N_2_ + CH_4_) ratio of 0.90. As reported by Kamal et al., the hydrophobic behavior of these films is generally attributed to surface roughness, morphology, and chemical bonding configurations [33]. Based on our results, it is clearly demonstrated that the main factor determining the wetting behaviors of these films is the presence of nanostructures in the film. Figure 9 shows a model representing the interaction of a water droplet with the surface of the film. For the film obtained at N_2_:(N_2_ + CH_4_) ratios of 0, 0.8, and 0.9, the contact area of the film–water interface is large, thus allowing the water droplet to spread on the film surface, resulting in low CA values. In contrast, the water droplet on the nanostructured film surface is suspended on the tips of the nanostructures due to trapped air within the nano-gaps in between the fiber strands, thus increasing the CA values. The results show that improvement in the alignment and uniformity of the nanostructures enhances the hydrophobicity of the films, as demonstrated by the films deposited at N_2_:(N_2_ + CH_4_) ratios of 0.7 and 0.75.

## 4. Conclusions

We have successfully deposited nanostructured carbon nitride films using a simple rf-PECVD technique at low temperature (100–220 °C) in a single step process without catalyst or template assistance. The effects of varying the N_2_:(N_2_ + CH_4_) ratio from 0.40 to 0.90 were studied. Fibrous nanorod structures were formed in the films deposited at N_2_:(N_2_ + CH_4_) ratios of 0.50–0.75. Uniform compact growth of vertically-aligned nanorod structures was achieved only in the films deposited within a narrow range of N_2_:(N_2_ + CH_4_) ratios of 0.70 and 0.75. These structures with high N/C ratio showed high aspect ratios, demonstrating that nitrogen incorporation was a crucial factor in the successful growth of these fibrous nanorod structures. Raman analysis indicated that the presence of the vertically-aligned nanostructures reduced residual stress in the lattice structure due to a decrease in defects in the film structure. The AES analysis showed that film was super-saturated with sp^2^ bonds, and the fibrous nanorod structures were actually precipitations of sp^2^ bonds in the film structure protruding out from the surface. The fibrous nanorod structures were formed as a result of the clustering of small graphitic sp^2^ clusters embedded within the amorphous matrix of the fibers. FTIR spectroscopy analysis showed a strong relationship between the formation of vertically-aligned nanorod structures with the formation of isolated and/or fused aromatic rings bonded to isonitrile bonds. The partial charge (−N^+^≡C^−^) formed within the nanostructure fibers contributed to aligned growth of the vertical nanorods, as a result of interaction between these polarized bonds and the electric fields produced by the rf plasma. The surface of carbon nitride films with vertically aligned fibrous nanorod structures was also hydrophobic with large contact angles of 131.7° and 125.5°.
